# Communication strategies to improve older adults’understanding of health information

**DOI:** 10.1590/0034-7167-2024-0083

**Published:** 2025-09-12

**Authors:** Juliana Piveta de Lima, Jamila Geri Tomaschewski Barlem, Daiane Porto Gautério Abreu, Rosemary Silva da Silveira, Marlise Capa Verde Almeida de Mello, Cenir Gonçalves Tier, Silomar Ilha

**Affiliations:** I Universidade Federal de Rio Grande. Rio Grande, Rio Grande do Sul, Brazil; II Universidade Federal do Pampa. Uruguaiana, Rio Grande do Sul, Brazil; III Universidade Federal de Santa Maria. Palmeira das Missões, Rio Grande do Sul, Brazil

**Keywords:** Health Communication, Health Literacy, Primary Health Care, Aged, Nursing, Comunicación en Salud, Alfabetización en Salud, Estrategia de Salud Familiar, Anciano, Enfermería

## Abstract

**Objectives:**

to identify communication strategies suggested by Primary Health Care professionals to enhance older adults’ understanding of health information.

**Methods:**

this qualitative, exploratory, and descriptive study involved 20 PHC professionals from the Health Units in a city in Rio Grande do Sul. Data were collected in June 2023 through focus groups and analyzed using discursive textual analysis.

**Results:**

the category Strategies that Facilitate Communication with Older Adults emerged from the analysis. This category included strategies such as group meetings, using drawings to facilitate understanding, limiting the amount of information provided in each consultation, speaking slowly, using simple language, repeating information, and applying the re-teaching technique.

**Final Considerations:**

professionals need training and guidance on communication strategies that can facilitate older adults’ understanding of health information.

## RESUMEN

**Objetivos:**

identificar estrategias de comunicación sugeridas por los profesionales de la Atención Básica, con la finalidad de mejorar la comprensión de las informaciones sobre la salud de las personas mayores.

**Métodos:**

investigación cualitativa, exploratoria y descriptiva realizada con 20 profesionales de la atención básica en las Unidades de Salud, de un municipio del estado de Rio Grande del Sur. Los datos fueron recopilados en junio de 2023, por medio de grupo focal y analizados por medio de análisis textual discursivo.

**Resultados:**

en el análisis surgió una categoría; la estrategia facilitadora de la comunicación con personas mayores, en la cual fueron citados recursos como la formación de grupos, la comunicación por medio de diseños, la limitación de las informaciones por encuentro, el hablar lentamente, el usar lenguaje simple, el repetir informaciones y la técnica de re-enseñanza.

**Consideraciones Finales:**

los profesionales necesitan de perfeccionamiento e informaciones sobre las estrategias de comunicación utilizadas para facilitar la comprensión de las informaciones por las personas mayores.

## INTRODUCTION

Functional Health Literacy (FHL), a social determinant of health, refers to an individual’s ability to understand, interpret, and critically reflect on health and disease-related information. Low FHL levels among older adults are associated with unfavorable health outcomes, including more extended hospital stays, lower health status, and higher mortality rates; older adults tend to use healthcare services more frequently, and their treatment outcomes are often less satisfactory^(1,2)^.

Research on FHL among older adults shows that this population often faces complex health conditions and struggles to understand and follow medical guidance regarding their clinical status, treatments, and medications^(1,3)^. These challenges affect self-care capacity and independence, leading to dissatisfaction with healthcare services and lower health outcomes^(1)^.

Primary Health Care and Family Health teams are responsible for the initial approach and multidimensional assessment of older individuals within their territories. Therefore, professionals must be able to assess and identify this population’s functional health status and provide specialized care when necessary^(4)^.

FHL experts recommend several actions and strategies to improve communication with patients who struggle to understand health information^(2)^. They emphasize the importance of training health professionals to make better use of their knowledge and skills, allowing them to coordinate care more effectively. To ensure comprehensive, high-quality care, professionals should also take into account each patient’s individual needs^(5)^.

In this context, the American Medical Association (AMA) and health education experts recommend a 14-item instrument to assess the use and effectiveness of communication techniques between patients and providers. These techniques include speaking slowly, using simple language, limiting information to two or three key points at a time, and confirming understanding through the re-teaching method^(6)^. Although studies show the effectiveness of these communication strategies^(7,8)^, most health professionals remain unaware of their importance and the negative implications for patients caused by a lack of understanding of health information, as well as the impact of low FHL on health outcomes.

Since health professionals are responsible for providing information and direct care to patients, they must be trained in communication techniques and understand the importance of identifying individuals with low FHL. Additionally, they should be aware of the specific considerations involved in communicating with older adults. This study aims to encourage discussion and reflection on the communication strategies used by health professionals and the implications of inadequate comprehension of health information. Based on this discussion, the following guiding question is proposed: What communication strategies do primary care professionals use to enhance older adults’ understanding of health information?

## OBJECTIVES

To identify communication strategies recommended by Primary Healthcare Professionals to enhance older adults’ understanding of health information.

## METHODS

### Ethical aspects

The Institutional Review Board at the Federal University of Rio Grande (CEP-FURG) approved this study. The participants received clarification regarding the study objectives and signed two copies of a free and informed consent form. Participants are identified by a letter and a number in each statement (e.g., HC1 for Health Professional 1, HC2 for Health Professional 2, and so on) to ensure their identities’ confidentiality. This study rigorously complied with ethical aspects provided for by law.

### Study design

This is a qualitative, exploratory, and descriptive study. The Consolidated Criteria for Reporting Qualitative Research (COREQ)^(9)^ were used to report the results.

### Methodological procedures

####  Study setting 

This study was conducted in the seven Family Health Units located in a town in Southern Rio Grande do Sul, Brazil. This town was chosen due to its low literacy rate-approximately 68% of the population has no formal education or has not completed primary education-and its low average monthly income (2.7 times the minimum wage)^(10)^, both of which are factors associated with low FHL levels.

####  Data source 

Primary health care (PHC) professionals participated in the study. The inclusion criteria required participants to be a nurse, physician, nursing technician, or community health agent (CHA) and to have worked at the unit for at least three months to ensure they had experience with the communication process. Professionals on sick leave and three physicians who had not met the minimum three-month work requirement were excluded.

####  Data collection and organization 

Data were collected using the focus group technique. Focus groups were conducted in June 2023 as part of the dissertation *Comunicação entre profissionais da saúde e pessoas idosas na atenção básica: estratégias para o Letramento Funcional em Saúde* [Communication between health professionals and older people in primary care: strategies for Functional Health Literacy]^(11)^. Each session lasted an average of 37 minutes.

The data were recorded and later transcribed into Word documents. Given their experience, the study’s author (an RN with a Master’s in Nursing) and an RN coordinated data collection, with the former serving as the mediator and the latter as the observer. The observer was trained through a thematic and methodological study group and received specific guidelines. One focus group was conducted with professionals working in the urban area, while another was held with professionals from the rural area. Both sessions were scheduled in advance by the primary care coordinator via telephone and took place at the town’s educational center, where the participating health professionals were present.

Focus groups are a form of group interview that relies on communication and interaction to gather in-depth information on a specific topic, aiming to understand beliefs, perceptions, and attitudes related to the subject under discussion^(12,13)^. The moderator facilitates the debate and ensures the session runs smoothly, while the quality of the information obtained depends largely on how the moderator conducts the discussion. Observers play a key role by monitoring and recording both verbal and nonverbal participant responses, assisting in the session’s facilitation, and managing time and recording equipment^(12,13)^.

Professionals from the seven ESF units (Bujuru, Carlos Santos, Cidade Baixa, Veneza, Tamandaré, Hélio Rossano, and Estreito) participated in the focus groups; 14 from the urban area and six from the rural area.

The focus group discussions were guided by the following question: “Which communication strategies are considered facilitators of communication with older adults?” The meetings explored strategies to improve health communication, the specific communication needs of the target audience, and the levels of FHL, including the assessed and related aspects. Additionally, the discussions incorporated data previously collected from older individuals and health professionals.

####  Data analysis 

Data were analyzed using the discursive textual analysis method^(14)^. After transcribing the focus groups, the texts were examined through detailed reading, with segments of similar meaning highlighted. This process led to the emergence of one category, in addition to strategies for facilitating communication with older adults, which provided insight into the phenomenon and allowed for identifying communication strategies suggested by primary care professionals to enhance older adults’ understanding of health information.

## RESULTS

The FHL level of the older adults interviewed was assessed, and all were found to have inadequate or low FHL. While the interviews with older adults did not reveal difficulties in understanding health information, health professionals reported that older individuals often struggle to comprehend the information provided during consultations. Consequently, this lack of understanding leads to poor adherence to treatments and health guidelines, ultimately resulting in poorer health outcomes. The focus groups were conducted with this issue in mind, aiming to identify communication strategies currently used by professionals, as well as potential strategies to improve older adults’ understanding of health information in the future.

The following category emerged from the analysis: strategies that facilitate communication with older adults (Chart 1).

**Chart 1:** Units of meaning and category, Rio Grande, Rio Grande do Sul, Brazil, 2023

Strategies to facilitate communication with older adults
- working with groups; - using drawings to facilitate communication; - limiting the amount of information provided per consultation; - speaking slowly, using simple language, and confirming whether the patient understood the instructions; - repeating information; - speaking loud; - asking for a family member to attend the consultation; - re-teaching technique; - establishing bonds with patients.

### Strategies that facilitate communication with older adults

The professionals reported forming groups to encourage experience sharing, which helps improve understanding of information and adherence to health guidelines.

*I am a person who strongly believes in groups* [...]*. If an older adult hears from someone next to him-someone going through the same situation-they might realize there is a consensus. And if someone else is doing it, they may think, ‘I will too’.* (HC1)

*Because there, they exchange information. Sometimes one participant asks a question that someone else was also wondering about, so it becomes easier to explain things using their language.* (HC2)

The professionals also mentioned using drawings as a strategy to help patients understand their medication schedules.

*They arrive at the clinic completely lost, carrying many medications but not knowing when to take them. We draw on the box-marking the medications for daytime and nighttime-to help them. It’s very complicated because many of them live alone.* (PS3)

*When they came to learn how to apply insulin, I gave them a pamphlet with a lot of written information and pictures to help them understand. There were images showing where to inject, how to store it in the refrigerator because I’m not sure they fully understand written instructions. So, I write everything down, and in addition to my explanation, I include pictures on the back.* (PS9)

Limiting the amount of information given during each consultation was another strategy mentioned by the participants.

*There’s no point in giving too much information because it will only confuse them, and they won’t understand. So, we have to provide just a little at a time.* (HC15)

The professionals also highlighted strategies such as speaking slowly, using simple language, and confirming the patient’s understanding, which they considered essential when providing health guidance.

*We already use simple language, speak slowly, and ask if they understood and how they are going to do it. We make sure they really got it.* (HC2)

*We use everyday language so they can understand. Sometimes, we mention things they are not familiar with, so I believe everyone tries to use simple terms and words to help them understand better.* (HC15)

Professionals reported that they typically repeat the information given during consultations.

*I repeat instructions more than once. I talk, explain, and then repeat so they can understand, because sometimes they don’t get it the first time.* (HC16)

Professionals reported that they often speak loudly to older adults.

*Another thing is speaking in a loud voice.* (HC15)

*I’m always shouting-I’m already going deaf.* (HC16)

When they notice that an older adult does not understand the information, professionals usually call a family member to assist.

*We say, ‘It’s easier to reach us. Tomorrow, if you can, come back with a family member’.* (PS4)

*If they don’t understand, we call a family member, or the CHA visits their home and asks a family member to explain. This way, the family member can guide the older adult or help them when it’s time to take their medication.* (HC15)

Another strategy used by professionals is asking the older adult how they will manage their treatment at home and having them explain it back. This technique, known as re-teaching, helps ensure understanding.

*I use this strategy-I explain it several times and then ask him how he’s going to take the medicine. He explains it to me, so I can be sure he understands.* (PS7)

Professionals also believe that establishing a bond of trust with older adults is essential.

*First, you have to build a bond. The patient needs to trust the professional providing care. Once they trust you, they start listening to your advice and eventually follow it.* (HC18)

## DISCUSSION

The literature shows that many patients do not understand health information and are unaware of their lack of understanding^(15)^. Additionally, evidence suggests a strong association between health professionals’ communication skills and the quality and effectiveness of health treatment^(16)^.

The PHC participants mentioned strategies such as forming groups to implement educational health actions. These activities take into account patients’ individual needs, customs, and beliefs^(17)^. In these meetings, different experiences are shared, allowing participants to exchange insights about diseases and living conditions, fostering ways to overcome health challenges^(6)^. Groups serve as an important resource in healthcare, as they are positively perceived by users and contribute to satisfactory therapeutic outcomes^(17)^.

Another strategy mentioned was drawing to help older adults understand their medication schedules. The decline in visual acuity with age can make reading information on medication packaging and prescriptions difficult, leading to errors or confusion in medication administration, particularly when drugs have similar names^(18)^. Drawings can aid in interpreting written instructions. Visualization tools like images, drawings, or graphs can improve patient understanding, especially when illustrating risks and benefits^(1,2)^.

Because complex information is difficult to understand, retain, and follow, it should be simplified and presented in steps, often across multiple consultations. The key points covered in a consultation should be limited, avoiding unnecessary details, as patients may lack the clinical knowledge to determine the most important information^(7)^. By narrowing the focus of each clinical encounter, understanding improves among patients with low FHL levels^(7)^.

Studies also show that using inappropriate, unnecessary, and undefined technical terms hinders patients’ understanding and adherence to care plans. Therefore, it is essential to adapt language to the target audience’s context^(7)^. Simple language should be clear and direct, avoid complex vocabulary, and facilitate message comprehension^(7)^. Since patients are often unfamiliar with technical terms, language should be adjusted whenever possible to align with their needs. Additionally, verifying the connection between patient understanding and information retention is important. One of the goals of Healthy People^(19)^ is to increase the proportion of professionals who ask patients to explain how they will follow instructions at home.

Furthermore, the aging process affects cognition, leading to changes in the speed and ability to process and retain information. Additionally, older adults are more prone to distraction during consultations. Therefore, essential information should be repeated, and reminders such as leaflets and notes should be used to support memory and recall, emphasizing key information that older patients need to follow later^(20)^.

Another important factor is age-related hearing loss, which leads to communication difficulties, isolation, and cognitive decline^(21)^. Hearing loss, or presbycusis, can prevent older individuals from fully participating in society, making it one of the most disabling communication disorders^(22,23)^.

To enhance communication with older adults, it is essential always to speak face-to-face, use a louder and slower speech pace, avoid covering the mouth^(22)^, and not accept hearing loss as a natural part of aging^(24)^. Since most older adults can benefit from hearing aids^(25)^, health professionals must take proactive steps to minimize the impact of hearing loss on quality of life^(24)^. A study conducted in Ireland on communication challenges for older adults highlighted the need for health professionals to recognize the effects of presbycusis on communication and the effectiveness of strategies such as repetition, reformulation, and noise reduction in improving health communication^(26)^.

The strategy of involving a family member during consultations is relevant, as family members are typically aware of the older adult’s health status and life circumstances, providing support and ensuring their needs are met. Family members play a crucial role in the care of older patients, as greater adherence to medication and better health habits are observed among those whose family members are actively involved in their care^(27,28)^.

Furthermore, patients rarely disclose whether they have understood the information provided, and one way to ensure comprehension is through the re-teaching method^(29)^. This approach involves asking patients to repeat the instructions health professionals give to assess their understanding, as comprehension of health information significantly impacts the adoption of positive health behaviors and outcomes. The re-teaching method has been widely used in health education programs and interventions^(15)^, as evidenced by a study conducted in the Netherlands with 396 health professionals, in which 99% of respondents recommended the method to improve patient understanding^(2)^.

Additionally, building a strong interpersonal relationship with patients is essential for effective communication. A patient-healthcare professional relationship based on trust increases patients’ confidence in their providers, helps them feel they are receiving the best care, enhances treatment satisfaction, and ultimately leads to better health outcomes^(30)^.

### Study limitations

The high demand for services at the health unit where the interviewed professionals work may have contributed to a lack of interest in participating in the study or led them to provide brief responses to the questions.

### Contributions to the Nursing field

Nurses who provide knowledge should be trained and informed about communication strategies to enhance patients’ understanding of health information. Older adults with low FHL, in particular, face more significant challenges in comprehending health information, and this lack of understanding directly impacts treatment adherence, medication use, and self-care. This study also contributes to practice and research by supporting the development of appropriate communication strategies that consider the specific needs of older adults to improve care for this population.

## FINAL CONSIDERATIONS

Professionals mentioned various strategies to facilitate effective communication with older patients, including forming groups, using drawings, limiting the amount of information per consultation, speaking slowly, using simple language, repeating information, speaking loudly, requesting the presence of a family member during consultations, applying re-teaching techniques, and establishing bonds with patients.

## Data Availability

Not applicable.
