# Supplementary material for: Communication strategies to improve older adults’understanding of health information
Source: Rev Bras Enferm. 2025 Sep 12;78(Suppl 2):e20240083. doi: 10.1590/0034-7167-2024-0083 (PMC12431794; doi:10.1590/0034-7167-2024-0083)
Supplement: Translation

# Translation: Estratégias de comunicação para melhorar a compreensão das informações em saúde de pessoas idosas

**DOI:** 10.1590/0034-7167-2024-0083pt

**Authors:** Juliana Piveta de Lima, Jamila Geri Tomaschewski Barlem, Daiane Porto Gautério Abreu, Rosemary Silva da Silveira, Marlise Capa Verde Almeida de Mello, Cenir Gonçalves Tier, Silomar Ilha

**Affiliations:** I Universidade Federal de Rio Grande. Rio Grande, Rio Grande do Sul, Brasil; II Universidade Federal do Pampa. Uruguaiana, Rio Grande do Sul, Brasil; III Universidade Federal de Santa Maria. Palmeira das Missões, Rio Grande do Sul, Brasil

**Keywords:** Comunicação em Saúde, Letramento em Saúde, Atenção Primária à, Saúde, Idoso, Enfermagem

## Abstract

**Objetivos:**

identificar estratégias de comunicação sugeridas pelos profissionais da Atenção Básica a fim de melhorar a compreensão das informações em saúde das pessoas idosas.

**Métodos:**

pesquisa qualitativa, exploratória e descritiva realizada com 20 profissionais da atenção básica nas Unidades de Saúde de um município do Rio Grande do Sul. Os dados foram coletados em junho 2023 por meio de grupo focal e analisados por meio da análise textual discursiva.

**Resultados:**

na análise, emergiu uma categoria; estratégias facilitadoras da comunicação com pessoas idosas, na qual foram citados recursos como a realização de grupos, comunicar-se por meio de desenhos, limitar as informações por encontro, falar devagar, usar linguagem simples, repetir informações e a técnica de re-ensino.

**Considerações Finais:**

os profissionais precisam de aperfeiçoamento e informação a respeito das estratégias de comunicação utilizadas a fim de facilitar a compreensão das informações pelas pessoas idosas.

## INTRODUÇÃO

O Letramento Funcional em Saúde (LFS), além de ser considerado como um dos determinantes sociais da saúde, se refere à capacidade de entender, compreender e refletir criticamente sobre informações relacionadas à saúde e à doença. Níveis baixos de LFS em pessoas idosas estão associados a resultados de saúde desfavoráveis, incluindo aumento da hospitalização, pior estado de saúde e maior mortalidade, uma vez que a utilização dos serviços de saúde é mais elevada nesse grupo etário e os resultados dos tratamentos médicos são menos satisfatórios^(1,2)^.

Pesquisas anteriores sobre LFS com pessoas idosas demonstraram que esse grupo populacional muitas vezes vive com complexas condições de saúde e possui grandes dificuldades em compreender e seguir orientações a respeito de suas condições clínicas, tratamentos e instruções sobre medicamentos^(1,3)^. Esses resultados afetam a capacidade de autocuidado e independência, gerando insatisfação do usuário com o serviço e resultados ruins de saúde^(1)^.

As equipes da Atenção Básica e de Saúde da Família são responsáveis pela primeira abordagem e avaliação multidimensional das pessoas idosas dos territórios. Os profissionais devem saber avaliar e identificar as condições funcionais desse grupo populacional e, caso necessário, complementar o cuidado com atenção especializada^(4)^.

Tendo em vista a dificuldade de compreender as informações em saúde, a maioria dos especialistas em LFS enfatiza diversos comportamentos e estratégias vitais para promover uma comunicação clara^(2)^. De acordo com esses especialistas, os profissionais de saúde podem ser capacitados para fazer melhor uso de seus conhecimentos e habilidades e, assim coordenar de forma mais eficiente os cuidados a serem realizados. Nesse sentido, o profissional da saúde deve considerar as necessidades individuais de cada paciente a fim de proporcionar uma assistência integral e de qualidade^(5)^.

Nesse âmbito, a *American Medical Association* (AMA) e especialistas em educação em saúde recomendam o uso de um instrumento de 14 itens que avalia o uso e a eficácia das técnicas de comunicação entre paciente e provedor, quais sejam: falar devagar, usar linguagem simples, limitar a duas ou três informações por vez e confirmar a compreensão por meio do método de re-ensino^(6)^. Embora os estudos demonstrem a eficácia da utilização das estratégias de comunicação^(7,8)^, a maioria dos profissionais da saúde não tem conhecimento sobre essas estratégias e as implicações negativas no paciente causadas pela falta de compreensão das informações em saúde, bem como as consequências do baixo LFS nos resultados de saúde.

Como os profissionais da saúde são responsáveis por fornecer informações e cuidados diretos aos pacientes, eles precisam ser capacitados a respeito das técnicas de comunicação e da importância de saber identificar pacientes com baixo LFS, além de conhecer as especificidades na comunicação com pessoas idosas. A realização desta pesquisa pretende estimular discussões e reflexões sobre as estratégias de comunicação utilizadas pelos profissionais e as implicações resultantes da falta de compreensão das informações em saúde. Frente ao exposto, questiona-se quais estratégias de comunicação são utilizadas pelos profissionais da atenção básica a fim de melhorar a compreensão das informações em saúde das pessoas idosas?

## OBJETIVOS

Identificar estratégias de comunicação sugeridas pelos profissionais da Atenção Básica a fim de melhorar a compreensão das informações em saúde das pessoas idosas.

## MÉTODOS

### Aspectos éticos

Esta pesquisa foi aprovada Comitê de Ética em Pesquisa da Universidade Federal do Rio Grande (CEP-FURG). Os participantes foram informados acerca dos objetivos mediante leitura e explanação do Termo de Consentimento Livre e Esclarecido (TCLE), que foi assinado em duas vias. Os participantes foram codificados por meio da utilização de letra e número arábico em cada depoimento (PS1, Profissional da Saúde 1, PS2 e assim por diante), com o intuito de preservar e garantir o seu anonimato. Todos os aspectos éticos previstos na legislação foram mantidos durante a condução do estudo.

### Tipo de estudo

Trata-se de uma pesquisa qualitativa, exploratória e descritiva. O *Consolidated criteria for reporting qualitative research* (*COREQ* ^(9)^ foi utilizado para reportar os resultados do estudo.

### Procedimentos metodológicos

####  Cenário do estudo 

A pesquisa foi realizada nas sete Unidades de Saúde da Família em um município do sul do Rio Grande do Sul. Este município foi escolhido para a realização da pesquisa por apresentar baixo índice de alfabetização - cerca de 68% da população não possui instrução ou ensino fundamental completo - e renda média mensal baixa (2,7 salários-mínimos)^(10)^, fatores que estão relacionados a um baixo nível de LFS.

####  Fonte de dados 

Profissionais da saúde da Atenção Básica participaram da pesquisa. O critério de inclusão foi ser profissional enfermeiro, médico, técnico de enfermagem ou ACS trabalhando há no mínimo três meses na unidade, a fim de ter vivenciado o processo de comunicação. Profissionais em licença de saúde foram excluídos, assim como três médicos que não alcançaram o tempo mínimo de trabalho de três meses na unidade.

####  Coleta e organização dos dados 

Os dados foram coletados por meio da técnica de grupo focal. Os grupos focais derivam da tese “Comunicação entre profissionais da saúde e pessoas idosas na atenção básica: estratégias para o Letramento Funcional em Saúde”^(11)^, foram realizados em junho 2023, com duração média de 37 minutos.

Os dados coletados foram gravados e posteriormente transcritos em documentos *Word*. A coordenação foi composta por uma moderadora (autora da pesquisa, enfermeira e mestre em enfermagem) e uma observadora (enfermeira), considerando suas experiências prévias. A observadora foi preparada por meio de grupo de estudo temático e metodológico e orientações. Foi realizado um grupo focal com os profissionais da zona urbana e um grupo focal com os profissionais da zona rural. Estes grupos foram agendados previamente, por meio de contato telefônico com a coordenadora da Atenção Básica e realizados com os profissionais da saúde no polo educacional do município.

O grupo focal é uma forma de entrevista com grupos baseada na comunicação e interação, e tem como objetivo reunir informações detalhadas sobre um tópico específico a fim de compreender crenças, percepções e atitudes acerca deste tema^(12,13)^. O moderador é um facilitador do debate e tem papel significativo para o bom funcionamento do encontro. A qualidade das informações nos grupos focais está atrelada à forma como o moderador conduz as sessões. Os observadores acompanham e registram as expressões dos participantes (verbais e não verbais), auxiliam na condução dos encontros, e controlam o tempo e o equipamento de gravação^(12,13)^.

Profissionais representantes das sete unidades de ESF do município (Bujuru, Carlos Santos, Cidade Baixa, Veneza, Tamandaré, Hélio Rossano e Estreito) participaram dos grupos focais; 14 no grupo da zona urbana e seis no da zona rural.

Os grupos focais foram norteados e problematizados pela seguinte questão: “Quais estratégias de comunicação podem ser consideradas facilitadoras da comunicação com pessoas idosas?”. Estratégias para melhorar a comunicação em saúde, particularidades na comunicação que devem ser consideradas acerca do público-alvo, e o LFS (aspectos avaliados e fatores relacionados) também foram abordados nesses encontros, e retornados os dados referentes às entrevistas com as pessoas idosas e os profissionais da saúde realizadas previamente.

####  Análise dos dados 

A análise dos dados se deu por meio do método da análise textual discursiva^(14)^. Após a transcrição dos grupos focais, os textos foram analisados através de leitura minuciosa, destacando as unidades de sentido com significados semelhantes. A partir disso, emergiu uma categoria, estratégias facilitadoras da comunicação com pessoas idosas, a qual permitiu o entendimento do fenômeno estudado e a identificação das estratégias de comunicação sugeridas pelos profissionais da atenção básica, a fim de melhorar a compreensão de pessoas idosas a respeito das informações em saúde.

## RESULTADOS

O LFS das pessoas idosas entrevistadas foi avaliado e todas obtiveram LFS inadequado ou marginal. A partir das entrevistas realizadas com as pessoas idosas, foi referido que não há dificuldades na compreensão das informações em saúde. No entanto, ao entrevistar os profissionais da saúde, estes relataram que as pessoas idosas possuem dificuldades em compreender as informações prestadas durante os atendimentos e em consequência disso, possuem resultados ruins de saúde, uma vez que não conseguem aderir aos tratamentos e às orientações em saúde. Os grupos focais foram realizados nesse sentido, a fim de elencar as estratégias de comunicação utilizadas pelos profissionais, ou as que eles possam vir a utilizar com a população idosa de forma a facilitar a compreensão das informações em saúde.

Na análise, emergiu uma categoria: estratégias facilitadoras da comunicação com pessoas idosas, conforme Quadro 1.

**Quadro 1:** Unidades de sentido do estudo e categoria, Rio Grande, Rio Grande do Sul, Brasil, 2023

Estratégias facilitadoras da comunicação com pessoas idosas
- realização de grupos; - comunicar-se por meio de desenhos; - limitar informações por encontro; - falar devagar, utilizar linguagem simples e confirmar se paciente entendeu as informações; - repetir as informações; - falar mais alto; - participação do familiar no atendimento; - técnica do re-ensino; - estabelecimento de vínculo com o paciente.

### Estratégias facilitadoras da comunicação com pessoas idosas

A estratégia de realização de grupos foi destacada pelos profissionais. Segundo eles, há uma troca de vivências entre os participantes e isso facilitaria a compreensão e adesão das informações.

*Eu sou uma pessoa que aposto muito em grupos* [...]*. Se* [o idoso] *ouvir de uma outra pessoa que está do seu lado, vivenciando a mesma situação, pode ser que ele chegue em um consenso que ele fez assim, e eu vou fazer também.* (PS1)

*Porque ali eles estão tendo uma troca, às vezes um fala e era a dúvida de outro, e é mais simples explicar na linguagem deles.* (PS2)

A estratégia de comunicar-se por meio de desenhos também foi relatada pelos profissionais, pois facilitaria a compreensão dos horários dos medicamentos.

*No posto eles chegam muito perdidos, com muitas medicações que eles não sabem os horários. A gente tem tentado desenhar na caixinha o que é do dia e o que é da noite para tentar ajudar. É muito complicado, porque muitos são sozinhos.* (PS3)

*Quando eles foram lá aprender a fazer aplicação de insulina, eu entreguei um folheto com um monte de informação escrita e depois as figuras para eles entenderem, figuras de onde aplicar, figuras da geladeira, de onde armazenar, porque não tenho certeza se eles vão entender o que está escrito, eu coloquei tudo escrito certinho e atrás, além de eu explicar, atrás eu também botei as figuras.* (PS9)

Limitar as informações por encontro foi uma estratégia referida pelos profissionais.

*Não adianta dar um monte de informações, vai deixar a pessoa mais confusa, ela não vai entender, tem que ser pouca* [informação]. (PS15)

Estratégias como falar devagar, utilizar linguagem simples e confirmar se o paciente entendeu as informações também foram referidas pelos profissionais e julgadas como importantes durante suas orientações em saúde.

*A gente já utiliza a linguagem simples, falar devagar, perguntar se entendeu e como é que vai fazer, se entendeu realmente.* (PS2)

*A gente fala no popular, que eles entendam. Tem coisas que a gente vai falar e eles não sabem nem o que significa. Então acredito que todas falam mais no popular para eles entenderem melhor.* (PS15)

Os profissionais relataram que costumam repetir as informações prestadas durantes os atendimentos.

*Quando eu vou orientar, eu procuro repetir mais de uma vez. Falo, explico, torno a falar, para eles entenderem, porque às vezes eles não conseguem pegar no momento que a gente fala.* (PS16)

Os profissionais relataram que costumam adotar a estratégia de falar mais alto com os idosos.

*Essa questão da voz alta também.* (PS15)

*Eu estou sempre gritando, eu já sou surda.* (PS16)

Ao perceberem que o idoso não compreende as informações, os profissionais também referiram que costumam chamar um membro da família para auxiliá-los.

*É mais fácil a gente falar, amanhã, se puder, volte aqui com o familiar.* (PS4)

*Se eles não estão entendendo, chama um familiar ou a ACS vai na casa deles e chama um familiar na casa para poder explicar, para ele poder orientar o idoso ou ajudar ele na hora que tiver que tomar medicação.* (PS15)

Outra estratégia adotada pelos profissionais é perguntar como o idoso vai seguir o tratamento em casa e pedir para ele explicar, essa técnica é conhecida como re-ensino.

*Eu faço isso, eu explico várias vezes e depois pergunto para ele de que forma você vai tomar esse medicamento. Ele me explica, para eu ter certeza que ele entendeu.* (PS7)

Os profissionais também acreditam que na relação com o idoso é preciso criar um vínculo com confiança.

*Primeiramente tem que criar o vínculo, o paciente tem que confiar no profissional por quem está sendo atendido, depois que eles confiam, eles acabam escutando o que o profissional orienta e acabam fazendo.* (PS18)

## DISCUSSÃO

A literatura demonstra que grande parte dos pacientes costuma não compreender as informações em saúde e eles acabam não reconhecendo essa falta de compreensão^(15)^. Também há evidências da íntima associação entre as habilidades de comunicação dos profissionais de saúde e a qualidade e eficácia dos tratamentos de saúde^(16)^.

Nesse sentido, a estratégia de realizar grupos foi citada pelos profissionais entrevistados, por meio da qual são realizadas ações educativas em saúde na atenção básica. As atividades em grupo levam em consideração as necessidades de cada indivíduo, seus costumes e suas crenças^(17)^. As diferentes vivências são abordadas nestes encontros, a fim de trocar experiências sobre doenças e condições de vida, criando assim formas de superar os problemas de saúde^(6)^. Os grupos são um importante recurso na assistência, uma vez que tais práticas possuem uma percepção positiva dos usuários, além de resultados terapêuticos satisfatórios^(17)^.

Outra estratégia referida foi a utilização de desenhos para facilitar a compreensão dos horários dos medicamentos. A diminuição da acuidade visual com o avanço da idade pode ocasionar dificuldades na leitura das informações contidas na embalagem do medicamento e nas receitas médicas, resultando em erros ou confusão na administração dos medicamentos, principalmente daqueles com nome semelhante^(18)^, e a utilização de desenhos facilitaria a compreensão das informações realizadas por escrito. Ferramentas de visualização, como imagens, desenhos ou gráficos podem melhorar a compreensão do paciente, principalmente se abordarem riscos e benefícios^(1,2)^.

Como as informações complexas são dificilmente entendidas, lembradas e seguidas, elas devem ser simplificadas e divididas em etapas (mais de um encontro). Os pontos-chave abordados no encontro devem ser delimitados, evitando detalhes desnecessários, uma vez que os pacientes podem não ter experiência clínica para eleger quais informações são mais importantes do que outras^(7)^. Ao limitar o foco de cada encontro clínico, aumenta a compreensão de pacientes com baixo nível de LFS^(7)^.

Estudos também demonstram que o uso de termos técnicos inadequados, desnecessários e sem definição prejudicam a compreensão e a adesão dos pacientes aos seus planos de cuidados. Portanto, é importante adaptar a linguagem para a realidade do público-alvo^(7)^. A linguagem simples deve ser clara e direta, evitar vocabulário complexo e facilitar o entendimento da mensagem pelo público-alvo^(7)^. Como os pacientes não costumam conhecer termos técnicos, deve-se utilizar linguagem simples e adaptada, sempre que possível, à realidade do paciente que está sendo atendido. Além disso, também é importante verificar a associação entre a compreensão dos pacientes e uma maior recordação das informações. Um objetivo da Health Peolple^(19)^ é aumentar a proporção de profissionais que pedem para descrever como os pacientes seguirão as instruções em casa.

Ademais, o processo de envelhecimento afeta a cognição, de maneira que ocorrem alterações na velocidade e capacidade processar e lembrar das informações, além da maior tendência à distração do idoso durante os atendimentos. Por isso, as informações essenciais devem ser repetidas e utilizados lembretes que auxiliem na memória/lembrança (folhetos, anotações), enfatizando as informações julgadas como importantes que o idoso deverá seguir posteriormente^(20)^.

Outro ponto de destaque é a perda auditiva relacionada à idade que resulta em dificuldades de comunicação, isolamento e declínio cognitivo^(21)^. A perda auditiva ou presbiacusia impede o idoso de desempenhar plenamente seu papel na sociedade, tornando-se um dos distúrbios de comunicação mais incapacitantes^(22,23)^.

Para aprimorar o processo de comunicação com as pessoas idosas, é necessário falar sempre de frente, mais alto, devagar, evitar cobrir a boca^(22)^ e não considerar a perda auditiva como parte aceitável do envelhecimento^(24)^. Como a maioria das pessoas idosas pode ser beneficiada por aparelhos auditivos^(25)^, os profissionais da saúde devem agir com urgência, fim de reduzir o impacto da perda auditiva na qualidade de vida^(24)^. Estudo realizado na Irlanda para identificar os desafios de comunicação pelas pessoas idosas, demonstrou a necessidade de conscientização dos profissionais da saúde sobre o impacto causado pela presbiacusia na comunicação e a utilidade de estratégias como a repetição, a reformulação e a minimização de ruídos para melhorar a comunicação em saúde^(26)^.

A estratégia de chamar um membro da família durante as consultas é relevante uma vez que os familiares costumam ter conhecimento da situação de saúde e circunstâncias de vida da pessoa idosa, além de serem responsáveis por fornecer apoio e garantir que suas necessidades sejam supridas. Os membros da família desempenham importante papel no cuidado de pacientes idosos, visto que se percebe uma maior adesão no uso de medicações e melhores hábitos de saúde naqueles que possuem envolvimento do familiar nos seus cuidados^(27,28)^.

Além disso, raramente os pacientes revelam se compreenderam as informações que lhes foram apresentadas, e uma forma de garantir a sua compreensão é por meio do método de re-ensino^(29)^. Nesse método, o usuário é solicitado a repetir as instruções recebidas dos profissionais de saúde para avaliação de sua compreensão, pois entender as informações em saúde tem impacto significativo na adoção de comportamentos e resultados favoráveis em saúde. Esse método tem sido amplamente utilizado como parte de programas e intervenções de educação em saúde^(15)^, conforme demonstrado em estudo realizado na Holanda com 396 profissionais da saúde, no qual 99% dos entrevistados recomendam o método a fim de melhorar o entendimento dos informações^(2)^.

Somado a isso, para uma comunicação eficaz, é necessário desenvolver um bom relacionamento interpessoal com o paciente. O relacionamento paciente-profissional da saúde baseado em confiança resulta em um crédito aumentado em seus provedores, além de fazer com que os pacientes acreditem estar recebendo os melhores cuidados, tornando-os mais satisfeitos com o tratamento e resultando em melhores resultados em saúde^(30)^.

### Limitações do estudo

A demanda dos serviços na unidade de saúde dos profissionais entrevistados pode fazer com que estes não tenham interesse em participar da pesquisa ou respondam de maneira suscinta aos questionamentos.

### Contribuições para a área da enfermagem

Os enfermeiros provedores de conhecimento precisam de aprimoramento e informação a respeito do uso de estratégias de comunicação para facilitar a compreensão das informações em saúde. Especialmente as pessoas idosas com baixo LFS possuem maior dificuldade em compreender as informações, e a não compreensão refletirá diretamente na adesão aos tratamentos de saúde, no uso de medicamentos e autocuidado. Esse estudo também causa impactos na prática e na pesquisa uma vez que, traz subsídios para o desenvolvimento de estratégias de comunicação adequadas que considerem as especificidades da pessoa idosa, visando a melhoria da atenção a este grupo populacional.

## CONSIDERAÇÕES FINAIS

Os profissionais referiram estratégias consideradas facilitadoras para uma boa comunicação com as pessoas idosas como a realização de grupos, comunicar-se por meio de desenhos, limitar informações por encontro, falar devagar, utilizar linguagem simples, repetir as informações, falar mais alto, participação do familiar durante o atendimento, técnica do re-ensino e estabelecer vínculo com o paciente.

## Data Availability

Não se aplica.
